# The Prebiotic-Like Effects of *Coprinus comatus* Polysaccharides on Gut Microbiota in Normal Mice and Those with Acute Alcoholic Liver Injury: A Comparative Study

**DOI:** 10.1155/2020/2027570

**Published:** 2020-11-28

**Authors:** Weidong Li, Yongxia Wang, Min Sun, Yuting Liang, Xiaoqing Cai, Dongmei Qi, Yongqing Zhang, Chunchao Han

**Affiliations:** ^1^School of Pharmacy, Shandong University of Traditional Chinese Medicine, Jinan 250355, China; ^2^Experimental Center, Shandong University of Traditional Chinese Medicine, Jinan 250355, China

## Abstract

This study aims to investigate the prebiotic-like effects of *Coprinus comatus* polysaccharides (CCP) on gut microbiota. Mice were divided into four groups: normal group (NG), alcohol group (AG), polysaccharides group (PG), and alcohol + polysaccharides group (APG). The gut microbiota structure of feces was analyzed by determining the V3-V4 region sequence in 16S rDNA. The results showed CCP could increase the diversity of gut microbiota. Compared with NG, PG had a significantly higher relative abundance of Firmicutes and Lactobacillaceae and a lower abundance of Rikenellaceae. These changes in gut microbiota result in positive effects on gut due to a series of prebiotic-like effects of CCP. At the same time, CCP could improve some adverse changes in gut microbiota caused by acute alcohol intake, such as the increased proportion of Firmicutes, Bacteroidetes, Muribaculaceae, and Lachnospiraceae and the decreased proportion of Rikenellaceae. In conclusion, the CCP has certain prebiotic effects not only on normal mice but also on mice with acute alcoholic liver injury.

## 1. Introduction

The consumption of alcohol has been rising with the development of the economy, which leads to a massive increase in the number of alcoholic liver injury cases. Patients with alcoholic liver injury frequently develop into liver disease such as liver fibrosis and cirrhosis, which could increase the risk of cancer [1]. The liver is also the organ most closely associated with the gut, and various liver diseases have been proved to be related to the altered gut microbiota [2].

Gut microbiota plays an important role in disease such as obesity, inflammatory bowel diseases (IBD), and liver cancer [3]. As an endocrine system, the gut microbiota communicates with other organs through a bidirectional communication system [4]. These communications are crucial to safeguarding the function of body, such as metabolic reactions, immune regulation, and pathogen elimination [5]. Hence, researchers paid more attention to designing and developing some innovative interventions to modulate gut microbiota, which could promote beneficial microbial-host interactions. Although the age, sex, and genes are important factors which are difficult to be changed, external environment could directly influence the gut microbiota such as prebiotics.

Research in the area of prebiotic interventions has increased exponentially in recent decades. As defined by the International Scientific Association, prebiotics are healthful substrates selectively used by host microorganisms [6]. The *Coprinus comatus* (*C. comatus*, Agaricaceae, and Agaricomycetes) is a new type of edible fungus in the north of China, which is used for food and medicine. *C. comatus* has some high nutritional ingredients, including polysaccharides, polyphenols, fatty acids, tocopherols, and organic acids. Especially, *C. comatus* polysaccharide (CCP) has been proven to contain activities, including antioxidation, hypoglycemic effect, and protection of the liver [7–9]. However, there are few studies on the probiotic-like effects of CCP on gut microbiota. Therefore, our experiment aims to compare the prebiotic-like effects of CCP in normal mice and those with acute alcoholic liver injury to observe the effect of CCP on gut microbiota.

## 2. Materials and Methods

### 2.1. Extraction of Polysaccharides from *C. comatus*

The fruiting body of *C. comatus* was provided by Jiangsu Chenxi Biotechnology Co., Ltd. (Jiangsu China). Polysaccharides fractions were extracted stepwise following the method of Li et al. [10] with some modifications. Dried in the oven (45°C), *C. comatus* was extracted twice with distilled water according to the ratio of 1 : 2 at 90°C for 3 h. After cooling to room temperature, the extract was mixed and centrifuged to obtain supernatants. The supernatants were concentrated to 1/4 of the original volume in the rotary evaporator. Then, the concentrated solution was mixed with absolute ethyl alcohol at 4°C overnight. Subsequently, through centrifugation at 4000 rpm for 15 min, the precipitate was obtained and washed with absolute ethyl alcohol and acetone consecutively. Redissolved in distilled water, the washed precipitates were mixed with a quarter volume of Sevag Reagent (Tianjin Fuyu Fine Chemical Co., Ltdj) to remove the proteins. The aqueous phase was then mixed with absolute ethyl alcohol at 4°C overnight to obtain the precipitation through centrifugation. After washing with acetone and petroleum ether, the precipitation was redissolved in distilled water and lyophilized to obtain the purified *C. comatus* polysaccharides.

### 2.2. Animal

Kunming (KM) mice were purchased from the Beijing Vital River Laboratory Animal Technology Co., Ltd. (Beijing, China). All of the mice were male, 4-5 weeks old, and weighing 20 ± 2 g. Standards for Animal Experimentation of Shandong University of Traditional Chinese Medicine were used in all experiments, which were in conformity to the rules of the Animal Ethics Committee of the institution (Shandong, China). The mice adapted the environment for seven days adaptively before the formal experiment. The states of the living environment are listed as follows: the living environment was specific pathogen free (SPF) grade. We maintained a schedule under a condition of 12/12 h light/dark. The mice room temperature was maintained at 18–20°C with a relative humidity of 40% to 60%. Animal feeding was according to the standard protocol. The room was ventilated on time, and all mice received humane care.

### 2.3. Experimental Design

After adapting the environment for 7 days, the mice were divided into four groups: NG (normal group), AG (alcohol group), PG (polysaccharides group), and APG (alcohol + polysaccharides group) (*n* = 10 per group). The PG and APG were orally administered by CCP (200 mg per kg bw) for 30 days. The NG and AG were given the same volume of distilled water for 30 days intragastrically. On the thirtieth day, the mice in each group were fasted for 16 hours overnight before modeling. After fasting, the AG and APG were given 50% ethanol (10 ml per kg bw) intragastrically, and the NG and PG were given the same volume of distilled water. After 24 hours of acute alcohol modeling, the fecal samples of mice were collected by the sterile EP tube. All fecal samples were stored at −80°C. Blood samples were collected and centrifuged at 3000 rpm for 10 min to separate serum for further analysis.

### 2.4. Determination of Biochemical Indexes

Serum alanine aminotransferase (ALT) and aspartate aminotransferase (AST) were analyzed using commercial kits according to the manufacturer's protocols (Rsbio, Shanghai, China).

### 2.5. DNA Extraction and 16S rDNA Gene Sequencing

HiPure Stool DNA Kits (Magen, Guangzhou, China) were used for extracting microbial DNA according to the manufacturer's protocols. The V3-V4 regions of the 16S rRNA were amplified with primers (341F:CCTACGGGNGGCWGCAG and 806R:GGACTACHVGG-GTATCTAAT) [11]. Subsequently, purified amplicons were pooled in equimolar amounts, and paired-end was sequenced on an Illumina platform according to standard protocols described by Gene Denovo Biotechnology Co., Ltd. (Guangzhou, China). The raw reads were further filtered through FASTP and merged as raw tags using FLSAH [12, 13]. Chimeric sequences were identified and removed using UCHIME [14]. UPARSE pipeline was used for assembling the effective tags into operational taxonomic units (OTUs) of ≥97% similarity [15]. The taxonomical assignment of OTUs was performed by the RDP classifier algorithm against the Silva database [16]. The analysis such as species annotation, *α* diversity analysis, and *β* diversity analysis was carried out successively according to the analysis process of OTUs.

### 2.6. Statistical Analysis

All data are expressed as the mean ± SD (standard deviations). Significant differences between two groups were assessed by using the Student's *t* test. Comparison between multiple groups was analyzed by using the one-way ANOVA test followed by the Student–Newman–Keuls (SNK) test. Data were collected and analyzed using Statistical Product and Service Solutions (SPSS) 26.0 software (SPSS Inc., Chicago, IL, USA). *P* < 0.05 was considered statistically significant.

## 3. Results and Discussion

### 3.1. Biochemical Indicators of Liver Injury

**Figure 1:**
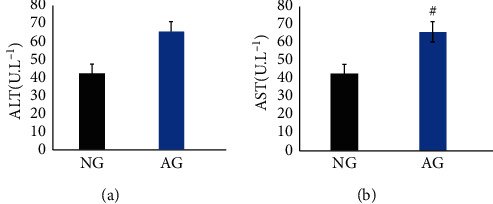
The effects of CCP on serum ALT and AST in mice. Values are presented as mean ± SD for 10 mice in each group. ^#^*P* < 0.05 as compared with the NG.

The serum ALT and AST levels, as biochemical indicators of liver injury, were significant increased in alcohol-treated mice (*P* < 0.05) (Figure 1).

**Figure 2:**
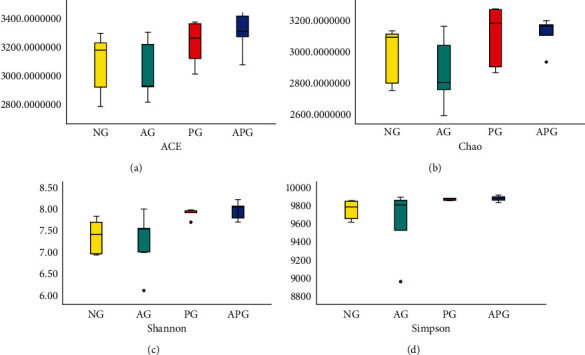
Differences in gut microbial diversity and richness of NG, AG, PG, and APG. (a) ACE; (b) Chao; (c) Shannon diversity; (d) Simpson diversity. ^#^*P* < 0.05 as compared with the NG.

The default provided 97% consistency to cluster the sequence into OTUs (Operational Taxonomic Units) with an average of 1999 per sample. Alpha diversity was used for reflecting richness and diversity of gut microbiota through ACE, Chao1, Shannon diversity index, and Simpson diversity index. The richness of gut microbiota (Figures 2(a) and 2(b)) in PG and APG was increased compared with NG, but there was no significant difference. However, the data of Shannon diversity (Figures 2(c) and 2(d)) indicated that the diversity of gut microbiota in APG and PG was significantly higher than NG (*P* < 0.05). Meanwhile, AG was not different from NG in the richness and diversity of gut microbiota according to Figure 2.

### 3.2. Composition of Gut Bacteria

**Figure 3:**
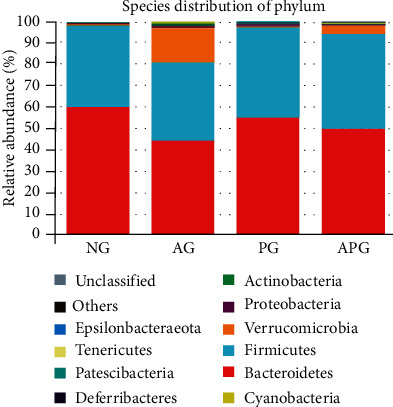
The relative abundance of gut microbiota at the phylum level.

At the phylum level (Figure 3), the two principal phyla of gut microbiota were Bacteroidetes (59.94%) and Firmicutes (37.73%) in the NG. Compared with NG, the proportion of Bacteroidetes was reduced by 15.77% in AG, 10.18% in APG, and 4.97% in PG. Compared with NG, the ratio of Firmicutes was increased by 6.58% in APG and 4.18% in PG, and decreased by 1.58% in AG. Furthermore, the relative proportion of Verrucomicrobia in AG was significantly higher than that of the other three groups (*P* < 0.05).

**Figure 4:**
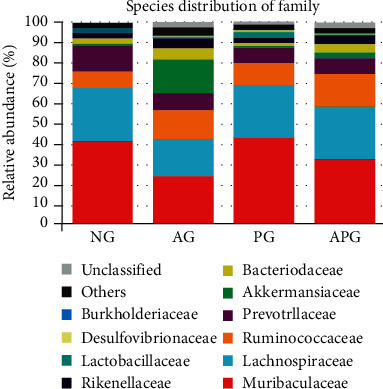
The relative abundance of gut microbiota at the family level.

At the family level (Figure 4), the two principal phyla of gut microbiota were Muribaculaceae (41.11%) and Lachnospiraceae (26.51%) in the NG. There was no significant difference in the proportion of Muribaculaceae between NG and PG. But compared with NG, the proportion of Muribaculaceae in AG and APG was reduced by 17.27% and 9.07% (*P* < 0.05). At the same time, the proportion of Lachnospiraceae in AG was decreased by 8.75% compared with NG (*P* < 0.05). But, there were no significant differences among the other three groups except AG. And, the content of Rikenellaceae was obviously different among AG (5.00%), APG (4.45%), NG (2.50%), and PG (2.30%) (*P* < 0.05). Furthermore, Lactobacillaceae of PG (3.09%) showed a significant increase compared with NG (1.87%), AG (0.67%), and APG (0.48%). Meanwhile, the proportion of Akkermansiaceae in APG (2.99%) was 4 times that of NG (0.74%) and 6 times of PG (0.45%), and alcohol intake induced an abnormal rise of Akkermansiaceae compared with other groups.

**Figure 5:**
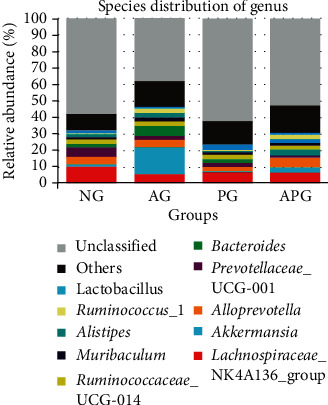
The relative abundance of gut microbiota at the genus level.

At the genus level (Figure 5), the content of *Akkermansia* in APG (2.99%) was 4 times that of NG (0.74%) and 6.5 times of PG (0.46%). Alcohol intake induced an abnormal rise of *Akkermansia* compared with other groups (*P* < 0.01). The populations of *Lactobacillus* in PG (3.08%) were significantly increased compared with NG (1.87%). But, alcohol intake could decrease the populations of *Lactobacillus* in AG (0.67%) significantly, which could not be improved in APG (0.48%) by CCP.

## 4. Discussion

The experiment aims to compare the prebiotic-like effects of CCP on gut microbiota in normal mice and those with acute alcoholic liver injury to explore the effect of CCP on gut microbiota. The results showed that the CCP could promote beneficial gut microbiota to some extent in normal mice and mice with acute alcoholic liver injury. However, the CCP could not adjust the gut microbiota of AG to normal state completely.

ALT and AST in serum are important indexes to evaluate liver injury. The previous studies have proven that patients with severe alcoholic liver injury present an increase in serum levels of ALT and AST in comparison with healthy individuals [17,18]. In the present study, ALT and AST levels of AG was increased significantly compared with those of NG (*P* < 0.05). Hence, our results proved the alcohol intake caused hepatotoxicity in the mice and the model of alcoholic liver injury was successful.

The alpha diversity (Chao, ACE, Shannon diversity, and Simpson diversity) of gut microbiota in APG and PG was increased compared with that of NG and AG, but only the significant differences of Shannon diversity was found in the data. We infer that the CCP could not increase the richness of gut microbiota, but it could improve the diversity of gut microbiota to some extent.

Bacteroidetes and Firmicutes were the two most abundant phyla among the all experimental groups. In previous studies, the changes in Bacteroidetes were not certain after chronic alcohol feeding. However, the proportion of Firmicutes was decreased under the same conditions [19–21]. Our results showed CCP could increase Firmicute level under normal feeding and alcohol intaking conditions. It has been reported Firmicute could produce more butyrate which is considered a health-promoting molecule. Meanwhile, CCP could improve the reduction in Bacteroidetes induced by alcohol. Bacteroidetes could produce more propionate to promote hepatic gluconeogenesis which could regulate the hypoglycemia caused by alcohol. Hence, we could preliminarily deduce the CCP could adjust the changes of two main phyla to produce prebiotic-like effects [22–24]. Verrucomicrobia was considered as a probiotic, which showed a negative correlation with hepatic inflammation and oxidative stress [25, 26]. Especially, Akkermansiaceae, the most representative gut microbe in the Verrucomicrobia, could prevent the weight gain caused by a high-fat diet, repair the damaged integrity of the intestinal epithelium barrier, reduce endotoxin levels in the blood, and improve insulin resistance [27, 28]. However, an interesting discovery we observed was that alcohol intake did not decrease the proportion of Verrucomicrobia but rather led to an obvious increase. At the same time, CCP had no significant effects on Verrucomicrobia under normal state. Through the analysis of individual data, we found that the abnormal increase of the Verrucomicrobia level was caused by one mouse in AG. By excluding the abnormal data, we found that CCP could inhibit the reduction of Verrucomicrobia caused by alcohol.

Muribaculacea played an important role in maintaining the gut balance. It could promote the production of the short-chain fatty acid (SCFA) [29], which can adjust the gut immune responses and protect the integrity of intestinal epithelial [30]. In our study, acute alcohol intake decreased Muribaculacea significantly, which could contribute to the destruction of normal intestinal function. However, CCP can significantly mitigate the adverse impact caused by alcohol, although it can not affect the proportion of Muribaculacea in normal state. Lachnospiraceae was another important bacterium for producing SCFA. The level of Lachnospiraceae was decreased in AG compared with NG, which could bring about adverse effects on the gut [31]. However, CCP could also alleviate the downward trend of Lachnospiraceae to ameliorate the adverse effects on the gut [32]. The previous study showed Nur77 knockout mice became obese accompanied with the relative abundances of increased Rikenellaceae [33]. At the same time, the abundance of Rikenellaceae was positively correlated with the hepatic inflammation [34]. In the current study, CCP could regulate Rikenellaceae properly to improve physical condition. Meanwhile, Lactobacillaceae could regulate the gut microbiota, enhance the immunity function, improve the gut function, and reduce the inflammation [35–37]. The proportion of Lactobacillaceae in PG was increased considerably compared with that in NG. Meanwhile, the same changes were observed in *Lactobacillus* at the genus level. Therefore, we inferred that CCP could promote the Lactobacillaceae level in the normal state.

## 5. Conclusions

In conclusion, the effect of CCP on gut microbiota was studied by comparing the prebiotic-like effects of CCP in normal mice and those with acute alcoholic liver injury. The prebiotic-like effects of CCP not only promote the health of gut under normal conditions but also improve some adverse changes in gut microbiota caused by acute alcohol intake. These functions are achieved by promoting beneficial microbiota and inhibiting harmful microbiota by CCP. Hence, we could further explore the development of CCP as a functional food for daily drinking to maintain a balance of gut microbiota. In addition, due to the complex relationship among dietary nutrition, gut microbiota, and host, uncovering specific effects of CCP on the gut still needs more evidence.

## Data Availability

The data used to support the findings of this study are included within the article.
